# JUVENILE XANTHOGRANULOMA: A CASE REPORT

**DOI:** 10.1590/1984-0462/;2019;37;2;00013

**Published:** 2019-02-25

**Authors:** Sara Pires da Silva, Catarina Viveiros, Rui Almeida, Marta Almeida Pereira, Rute Vaz, Alexandrina Portela

**Affiliations:** a Pedro Hispano Hospital, Local Health Unit of Matosinhos, Matosinhos, Portugal.

**Keywords:** Xanthogranuloma, juvenile, Histiocytosis, non-Langerhans-cell, Skin abnormalities, Xantogranuloma juvenil, Histiocitose de células não Langerhans, Anormalidades da pele

## Abstract

**Objective:**

To report a rate case of Juvenile xanthogranuloma in a newborn infant.

**Case description:**

We present the case of a 31-week preterm newborn with multiple skin lesions whose clinical, histological and immunohistochemical findings allowed the diagnosis of juvenile xanthogranuloma. Currently, the patient has nine months-old, and there is no aggravation of the skin lesions or evidence of extra-cutaneous involvement, particularly ophthalmic.

**Comments:**

Juvenile xanthogranuloma is a rare and benign condition, included in the vast group of non-Langerhans histiocytosis. It typically occurs in the pediatric age and may have a neonatal presentation. It affects predominantly the skin, in the form of papules or yellow and/or erythematous nodules and could be asymptomatic, multiple or solitary. Extra-cutaneous involvement, is more common in toddlers and when multiple lesions are present. The eye is the most affected site. We highlight this clinical case by its presentation in the neonatal period and in the form of multiple lesions, which bestows an increased risk of extra-cutaneous involvement, although this has not yet been verified.

## RESUMO

**Objetivo:**

Descrever um caso raro de xantogranuloma juvenil em recém-nascido.

**Descrição do caso:**

Apresentamos o caso de um recém-nascido pré-termo de 31 semanas com múltiplas lesões cutâneas cuja clínica, histologia e imuno-histoquímica permitiram o diagnóstico de xantogranuloma juvenil. Atualmente, com nove meses de idade, não apresenta agravamento das lesões nem evidência de envolvimento extracutâneo, nomeadamente oftálmico.

**Comentários:**

O xantogranuloma juvenil é uma patologia rara e benigna, pertencente ao vasto grupo das histiocitoses não Langerhans. Surge tipicamente em idade pediátrica, podendo ter apresentação neonatal. O envolvimento é predominantemente cutâneo sob a forma de pápulas ou nódulos de coloração amarela e/ou eritematosos, assintomáticos, solitários ou múltiplos. O envolvimento extracutâneo é mais frequente em crianças com menos de dois anos e com múltiplas lesões, sendo o olho o local mais afetado. Destacamos este caso clínico pela apresentação no período neonatal e sob a forma de múltiplas lesões, o que lhe confere risco acrescido de envolvimento extracutâneo, sem que, no entanto, tal se tenha verificado.

## INTRODUCTION

Juvenile xanthogranuloma (JXG) is a rare and benign proliferative disease, belonging to the vast group of non-Langerhans histiocytoses (NLH).^1^ ^,^ ^2^ ^,^ ^3^ ^,^ ^4^ ^,^ ^5^ ^,^ ^6^ ^,^ ^7^ First described by Adamson in 1905,^8^ its current nomenclature was adopted in 1954.^9^

With unknown etiology and incidence, it affects predominantly children in the first two years of life, and there has been cases of diagnosis in adulthood. ^1^ ^,^ ^2^ ^,^ ^3^ ^,^ ^4^ ^,^ ^5^ ^,^ ^6^ ^,^ ^7^ The usual presentation is cutaneous, with extracutaneous involvement being rare. ^1^ ^,^ ^2^ ^,^ ^3^ ^,^ ^4^ ^,^ ^5^ ^,^ ^6^ ^,^ ^7^ ^,^ ^10^ ^,^ ^11^ Diagnostic suspicion is clinically based and corroborated by histology and immunohistochemistry. ^2^ ^,^ ^3^ ^,^ ^4^ ^,^ ^5^ ^,^ ^6^

The purpose of this case report is to describe a rare case of juvenile xanthogranuloma in the neonatal period.

## CASE DESCRIPTION

Newborn male with gestational age of 31 weeks, born from a gestation that had prenatal follow-up and no intercurrences. Son of healthy, non-consanguineous parents. At birth, anthropometry and the objective examination were compatible with gestational age.

**Figure 1:**
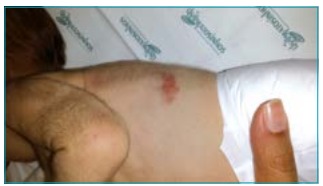
First lesion to appear, located on the left flank.

**Figure 2:**
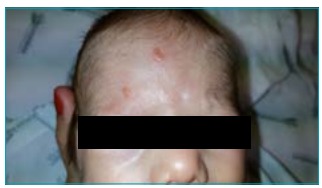
Lesions in the frontal region.

On the subject’s third day of life, an erythematous lesion with about 10 mm in diameter appeared on the left flank (Figure 1). Subsequently, similar lesions appear on the abdomen, back, hands, and chin. At the 30^th^ day of life, there were two nodular, firm, yellowish lesions with no inflammatory signs or ulceration, with 10 mm in diameter (Figure 2).

A cutaneous biopsy of the lesion located on the left flank was performed (Figure 1), whose histology revealed presence of histiocytes in the dermis, with no evidence of epidermotropism. The immunohistochemical study demonstrated positivity for cluster of differentiation (CD) 68 and negativity for CD1a, S100, HMB45 and ACL. These results confirmed the clinical diagnosis of NLH, namely JXG.

Faced with this result, the study was completed with transfontanelar, abdominal and bladder-kidney echography, which revealed no alterations. Ophthalmologic observation excluded the presence of ocular lesions.

Currently, the patient is 9 months old, the cutaneous lesions are stable and the evolution of weight and length is adequate. Regular neonatology, dermatology, pediatric hemato-oncology and ophthalmology follow-up in consultations was maintained.

## DISCUSSION

JXG is the most frequent form of NLH..^1^ ^,^ ^2^ ^,^ ^4^ ^,^ ^6^ Its etiology is unknown. Some authors postulate the existence of an alteration of the macrophagic response to a nonspecific stimulus such as trauma and viral infection, a hypothesis that still lacks evidence.^1^ ^,^ ^3^ ^,^ ^4^ ^,^ ^6^ ^,^ ^10^ ^,^ ^12^

Lesions appear in the first two years of life: 5 to 17% are present at birth and 40 to 70% appear during the first year.^1^ ^,^ ^2^ ^,^ ^6^ ^,^ ^7^ Its frequency is independent of race, but is higher in males (1.5:1.0).^2^ ^,^ ^3^ ^,^ ^4^ ^,^ ^7^

Because of its benignity and transitory character, it is estimated that it is an underdiagnosed entity and, therefore, of unknown incidence.^2^ ^,^ ^3^ ^,^ ^5^

The most frequent presentation is in the form of a solitary, exclusively cutaneous lesion. It appears as a well delimited, firm, elastic, rounded papule or nodule with 0.5 to 2.0 cm in diameter.^2^ ^,^ ^3^ ^,^ ^4^ ^,^ ^5^ ^,^ ^6^ ^,^ ^10^ Initially, it may present a reddish coloration, later evolving to a xanthomatous tonality, and sometimes superficial telangiectasias or erythematous halos are visible.^2^ ^,^ ^5^ ^,^ ^10^ Being asymptomatic, they appear more frequently in the head and neck, followed by the trunk and limbs, and may affect any point of the corporal surface, including mucous membranes.^2^ ^,^ ^7^ ^,^ ^10^ The form of multiple lesions occurs in 7 to 10% of the cases, predominating in infants aged less than 6 months, and in males.^3^ ^,^ ^5^ ^,^ ^7^ In this form, the lesions can appear and regress at different times, with old and recent wounds coexisting.^10^ Other more rare presentations are known, such as the giant form (with lesions larger than 2 cm), in aggregates, and in plaque or lichenoid form.^6^ ^,^ ^10^

Extracutaneous involvement is described, and is estimated to occur in 4% of cases, affecting any organ or tissue.^1^ ^,^ ^4^ ^,^ ^5^ Risk factors for extracutaneous involvement are age under two years and the presence of multiple lesions.^7^

Ocular lesions are the most frequent, occurring in 0.3 to 0.5% of cases. It is mostly unilateral, with the iris being the most affected site, followed by the eyelid. Orbital involvement is rare and predominates in the neonatal period.^2^ ^,^ ^4^ ^,^ ^5^ ^,^ ^10^ In half of the cases with ophthalmic involvement, there are no previous skin lesions..^1^ ^,^ ^5^ ^,^ ^7^ In the context of ocular involvement, the most frequent complications are hyphema, glaucoma, and anterior uveitis, with the risk of loss of visual acuity, which justifies a regular follow-up by ophthalmology, especially in patients with high risk.^2^ ^,^ ^5^ ^,^ ^6^ Ocular JXG is the most frequent cause of spontaneous hyphema in children.

The involvement of other organs such as the lung, liver, spleen, pericardium, bone, central nervous system, among others, with different systemic repercussions, is also described.^1^ ^,^ ^2^ ^,^ ^6^ ^,^ ^7^ The association of JXG to neurofibromatosis type I (NFI) and juvenile chronic myeloid leukemia (JCML) is documented.^1^ ^,^ ^2^ ^,^ ^4^ ^,^ ^5^ ^,^ ^6^ ^,^ ^10^ The concomitant presence of JXG and NFI brings an increased risk - 20 to 30 times - of developing JCML. ^2^ ^,^ ^4^ However, the association between JXG and Niemann-Pick disease, urticaria pigmentosa or cytomegalovirus infection lacks further clarification.^6^ ^,^ ^10^ Although the cutaneous lesions of JXG are typically xanthomatous, this pathology is not associated with changes in the lipidic or endocrine-metabolic profile..^2^ ^,^ ^6^ ^,^ ^12^

Diagnosis is fundamentally clinical. The differential diagnosis may include histiocytoses of Langerhans cells, Spitz nevi, urticaria pigmentosa, among others. In dubious cases, cutaneous biopsy should provide clarification. ^2^ ^,^ ^3^ ^,^ ^4^ ^,^ ^5^ ^,^ ^6^ In fact, the name “xantogranuloma” is due to its histological appearance, with histiocytes loaded with lipids, with vacuolated and xanthomatous cytoplasm.^2^ Histopathology shows typical histiocytosis findings, with frequent infiltration of the dermis, presence of multinucleated giant cells in variable numbers, and inflammatory cells in the perilesional areas. In 85% of cases, it is possible to observe Touton giant cells, resulting from the fusion of macrophages and characterized by a crown of nuclei, with homogeneous eosinophilic cytoplasmic center and prominent peripheral xanthomatization.^2^ ^,^ ^7^ ^,^ ^10^ Immunohistochemistry establishes the differential diagnosis with histiocytosis of Langerhans cells. Thus, for the diagnosis of JXG, the lesion cells will be positive for factor XIIIa, CD68, CD163, fascein and CD14, and negative for CD1a and S100, the latter being specific for Langerhans cells.^1^ ^,^ ^3^ ^,^ ^5^ ^,^ ^7^ ^,^ ^10^

At the time of diagnosis, a meticulous objective examination should be performed to determine the existence of systemic involvement. As extracutaneous involvement is rare, there is no consensus as to whether it should be excluded by analytical and/or imaging evaluation in the absence of suggestive signs or symptoms. Nevertheless, regular ophthalmologic assessment is recommended in children younger than two years of age and with multiple skin lesions.^2^ ^,^ ^4^ ^,^ ^5^ ^,^ ^10^

The prognosis of patients with exclusively cutaneous involvement is excellent, with spontaneous remission in months or a few years, and relapses are rare.^1^ ^,^ ^2^ ^,^ ^3^ ^,^ ^5^ ^,^ ^6^ ^,^ ^10^ In some cases, a small residual hyperpigmented scar may remain.^1^ ^,^ ^2^ ^,^ ^5^ ^,^ ^10^ Surgical removal may be considered only for cosmetic reasons, especially in cases of giant JXG.^2^ The cases with extracutaneous involvement present greater morbidity and mortality.^3^ Its therapeutic approach is not consensual and should be evaluated on a case-by-case basis. Therapeutic options include immunosuppression and chemotherapy, using corticosteroids, cyclosporine or methotrexate, radiotherapy and surgical excision, which should be considered casuistically.^5^ ^,^ ^6^

It can be concluded that JXG is a rare and benign disease, which typically occurs in pediatric age and is underdiagnosed. This clinical case was highlighted by the presentation in the neonatal period and in the form of multiple lesions, which gives it an increased risk of extracutaneous involvement, although this has not been verified. We emphasize the frequent need for histological confirmation and the importance of the ophthalmologic follow-up of these patients.
